# Damage Control Surgery for Duodenal Ulcer Bleeding With Massive Hematoma and Perforation Due to Over-the-Scope Clip (OTSC)

**DOI:** 10.7759/cureus.56359

**Published:** 2024-03-18

**Authors:** Kei Harada, Daibo Kojima, Ippei Yamana, Hirotaka Seike, Takahisa Fujikawa

**Affiliations:** 1 Surgery, Kokura Memorial Hospital, Kitakyushu, JPN; 2 Gastroenterological Surgery, Fukuoka University, Faculty of Medicine, Fukuoka, JPN

**Keywords:** emergency abdominal surgery, peptic ulcer bleed, duodenal ulcer disease, duodenal hematoma, over-the-scope clips, perforated duodenal ulcer, damage control resuscitation, damage control laparotomy

## Abstract

Due to the advances in endoscopic technology, surgery for duodenal ulcer (DU) bleeding has decreased, although surgery is still necessary for more complicated cases. The concept of damage control surgery (DCS) has been established in the field of trauma, and a simple surgical approach may be preferable in serious cases such as uncontrolled DU bleeding. We present a successful case of bleeding with massive hematoma and perforation of the duodenum due to an over-the-scope clip that was treated by a less invasive surgical approach with consideration of the DCS.

## Introduction

The frequency of surgery for peptic ulcer disease (PUD) has significantly decreased in the modern period due to the eradication of *Helicobacter pylori *and the development of anti-gastric acid medications like proton pump inhibitors [1,2]. However, surgery is still required in some cases, such as when it is difficult to stop bleeding with an endoscope, when the patient’s overall state is poor, or when complications arise as a result of endoscopic treatment [3,4].

Specifically, as we experienced, hemorrhagic shock-associated duodenal ulcer (DU) bleeding is a serious circumstance that requires immediate surgery, considering damage control surgery (DCS) [5,6]. DCS is a well-established surgical strategy for trauma treatment with hemodynamic stability as the primary goal and is now becoming popular in general emergency surgery [7].

Herein, we report a successful case of uncontrolled DU bleeding and perforation due to an over-the-scope clip (OTSC) that was treated with a less invasive surgical approach based on the concept of DCS. Furthermore, we hope that our case report will be useful in future treatment strategies for complicated cases of DU bleeding.

## Case presentation

**Figure 1:**
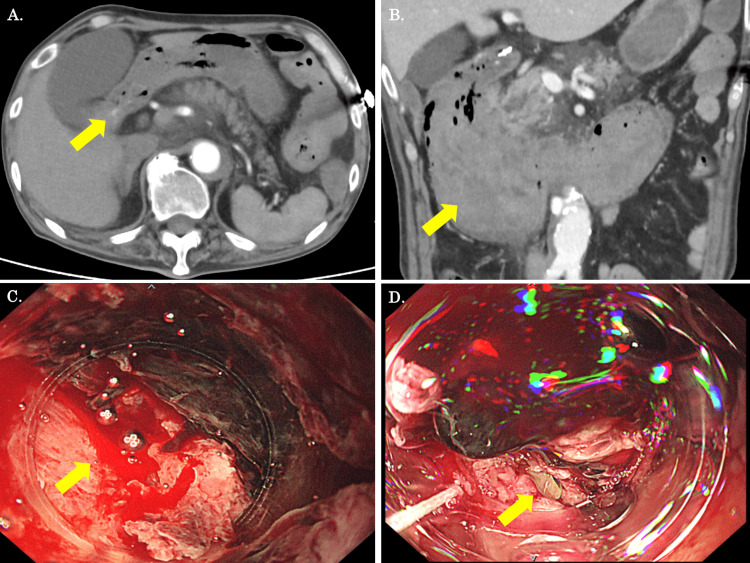
CT and EGD findings prior to the emergency operation (A, B) An enhanced CT scan showed that extravasation was present around the duodenal clipping point, and the duodenum was significantly dilated due to massive hematoma (yellow arrow). (C) An EGD showed that active bleeding was observed at the posterior lateral aspect of the duodenum (yellow arrow). (D) An EGD showed a mucosal defect that appeared to be perforation after OTSC (yellow arrow). EGD, esophagogastroduodenoscopy; OTSC, over-the-scope clip

An 80-year-old man was referred to the gastroenterology department for an esophagogastroduodenoscopy (EGD) after hematemesis on postoperative day (POD) 18 following an operation for acute type A aortic dissection (ATAAD). The patient had chronic atrial fibrillation and was on warfarin. In addition, he had not taken any peptic ulcer agents since the ATAAD operation, but he had been using non-steroidal anti-inflammatory drugs every day for pain management. The EGD showed a small ulcer and open blood vessels in the duodenal bulb, which was then clipped. Since there were no other lesions with active bleeding and the patient’s vital signs were stable, it was decided to perform conservative treatment and conduct a second look two days later. The next morning, the patient had hematemesis once more. An enhanced CT scan was conducted. The CT scan showed extravasation near the clip of the inferior wall of the duodenal bulb (Figure 1A). Furthermore, a massive hematoma was observed mainly in the duodenum (Figure 1B). At this time, the patient was referred to our surgical department. The vital signs recorded were as follows: blood pressure: 103/62 mmHg; pulse rate: 136 beats/min; and body temperature: 37.2°C. Blood gas analyses revealed the following values: pH: 7.26; partial pressure of arterial carbon dioxide: 89.1 mmHg; partial pressure of arterial oxygen: 152.7 mmHg; HCO3: 29.3 mmol/L; and lactate: 2.67 mmol/L. We opted to do an EGD in the ICU with the surgeons on standby. An EGD showed an ulcer on the lower wall of the upper intestinal angle. The ulcer was still oozing in one place (Figure 1C). A 9-mm OTSC was performed at the same location, and a mucosal defect that seemed to be a perforation was discovered at the base (Figure 1D).

**Figure 2:**
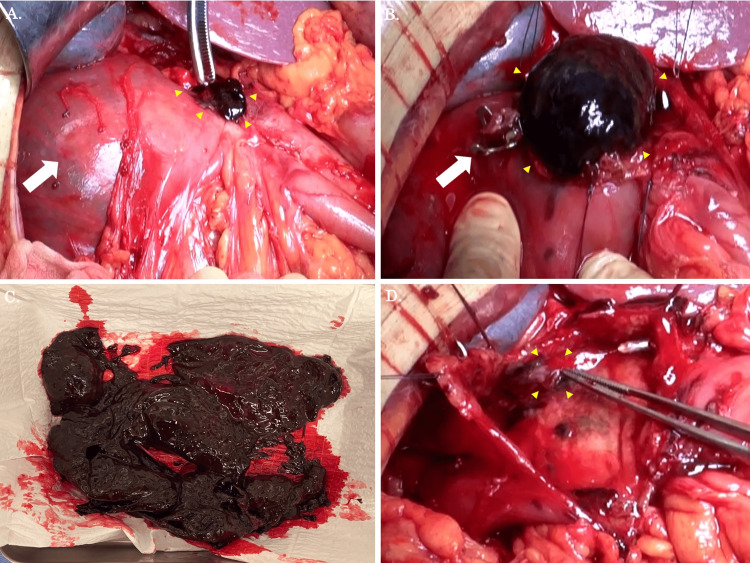
Intra-abdominal findings during operation (A) After a mobilization of the dilated duodenum (white arrow), the perforation site was observed (yellow arrows). (B) When the perforation site was opened to the gastric and duodenal sides, an OTSC (white arrow) and massive hematoma (yellow arrows) were found. (C) The hematoma removed was approximately 350 grams. (D) The bleeding from the wall of the duodenal ulcer, probably from a very small blood vessel distal to the gastroduodenal artery, from the duodenal wall (yellow arrows), was quickly halted with a suture. DU, duodenal ulcer; OTSC, over-the-scope clip

Despite blood transfusion therapy, shock vitals continued, acidosis worsened, and OTSC perforation was discovered. Therefore, we decided to perform emergency surgery, considering DCS. The surgical findings and methods are presented here. An incision was made in the midline of the upper abdomen under general anesthesia. After mobilization of the duodenum, a significantly dilated duodenum and a perforation site suspected to be caused by OTSC were detected (Figure 2A). We performed a longitudinal duodenotomy extending across the pylorus to the distal stomach, and then massive duodenal hematomas were successfully removed (Figure 2B). It was about 350 grams of hematoma that was removed (Figure 2C). The mucosal surface of the duodenum could be clearly observed, and the bleeding was quickly halted with a Z suture (Figure 2D).

**Figure 3:**
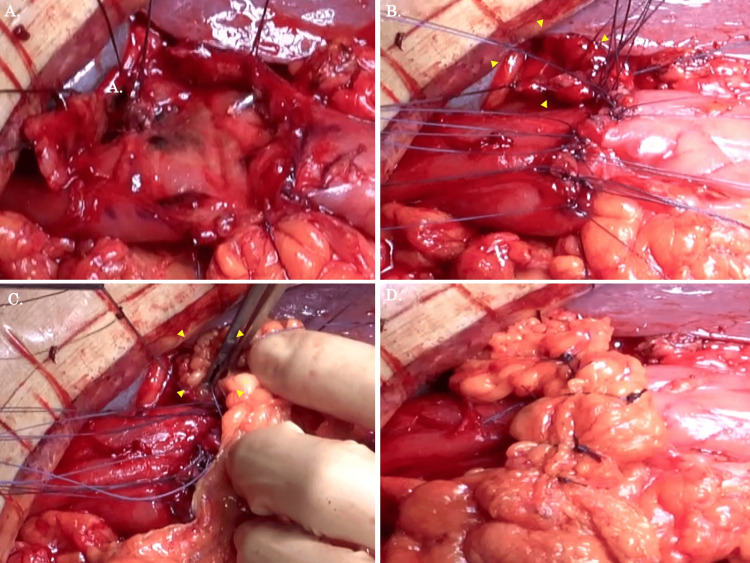
The surgical procedure is shown (A) After suturing the ulcer hemorrhage, the duodenal mucosal surface and tissue fragility were confirmed. (B) Interrupted suture closure was performed on the side where macroscopically normal duodenal mucosa remained. Suture closure was not performed in areas where the tissue was very fragile (yellow arrows). (C, D) Greater omentum filling and covering were performed in areas that were not closed with sutures (yellow arrows).

Based on an adaptation of the DCS idea, we decided that perforation closure and filling of the greater omentum were preferable to duodenal resection and gastrointestinal reconstruction. On the side where normal duodenal mucosa was still present, interrupted suture closure was carried out (Figure 3A, 3B). Greater omental filling was performed on a weak duodenal location with severe ulceration (Figure 3C). Additionally, the greater omental covering of the perforation was added with the thread left over after the interrupted suture closure (Figure 3D).

**Figure 4:**
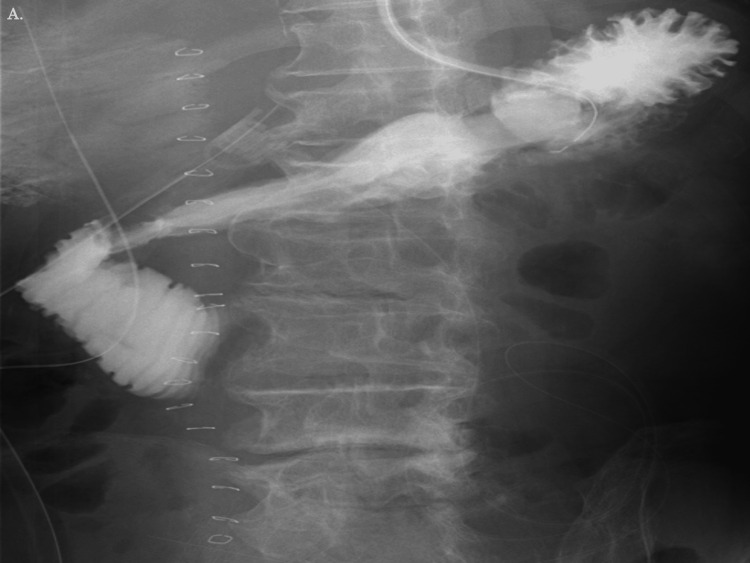
The oral gastrointestinal contrast examination findings on POD 9 The contrast medium flowed well, and no evident leaks or severe stenosis were found. POD, postoperative day

After that, a jejunostomy was made, and the operation was over. The time of the operation was two hours and 42 minutes, and 620 ml of blood was lost, including the hematoma that was removed. After the operation, the patient’s vitals returned, and acidosis improved. The patient was successfully extubated on POD 1, and enteral nutrition was started through the jejunostomy on POD 2. An oral intake was initiated on POD 9, following an oral gastrointestinal contrast test that verified no issues (Figure 4). Other than a little local intra-abdominal abscess and a urinary tract infection, the patient was able to eat normally after that and was sent to a rehabilitation facility without any major issues.

**Figure 5:**
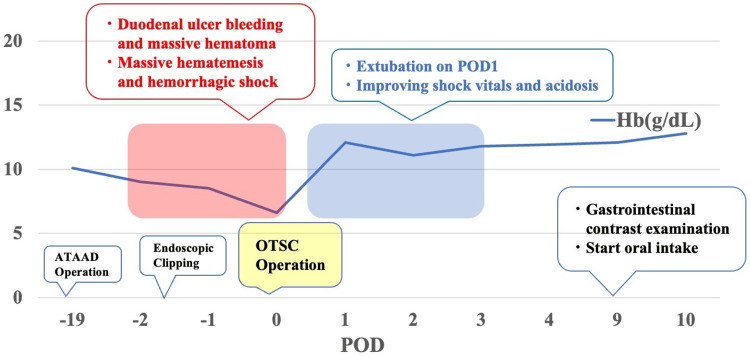
Clinical course of the patient ATAAD, acute type A aortic dissection; Hb, hemoglobin; OTSC, over-the-scope clip; POD, postoperative day The figure is the authors’ own creation.

Figure 5 summarizes the clinical course of the patient. In reporting the case, we respected ethical considerations, including the protection of personal privacy, and provided sufficient informed consent to the patient.

## Discussion

Upper gastrointestinal bleeding is still a medical condition requiring hospitalization, and peptic ulcer bleeding is reported to be the most common cause of bleeding, with a rate of 40-46% [8]. According to the treatment protocol for PUD, endoscopic hemostasis is the first line of treatment for bleeding ulcers. If this fails, surgery or interventional radiology is advised [1].

Especially for bleeding DUs, local methods (suture of ulcers with or without extraluminal gastroduodenal artery ligation) and gastroduodenal resections (distal gastrectomy with partial duodenectomy) are available. However, there is still a high rate of morbidity and mortality linked to surgery [9]. Endoscopic treatment for bleeding DU on the posterior wall of the duodenal bulb, as in our case, is difficult due to anatomical factors. Additionally, erosion of the gastroduodenal artery or its branches beyond the posterior duodenal wall can cause significant bleeding and increase the risk of rebleeding [10].

According to Kobara et al., an OTSC is a relatively safe device for an endoscopic procedure. However, all OTSC-related problems occurred in approximately 1.7% (26/1,517) of patients [11]. Additionally, there have also been reports of microperforation of bleeding DUs caused by OTSC [12]. In our case, we chose an OTSC as an endoscopic treatment before surgery to prevent rebleeding the following day after primary hemostasis. As a result, endoscopic hemostasis was incomplete, resulting in a poor blood transfusion response, leading to hemorrhagic shock, acidosis, and perforation associated with OTSC, which led to emergency surgery.

We adopted the concept of DCS to perform an emergency surgery. The aim of DCS is to lower the risk of death in seriously injured patients with physiological derangement, and its application has recently broadened to include bleeding from complex gastroduodenal ulcer illness, generalized peritonitis, acute mesenteric ischemia, and other diseases [13,14]. In our case, we performed an incision from the anterior wall of the duodenum to the gastric pylorus and sutures on the bleeding site with the aim of removing the extensive hematoma and stopping the ulcer bleeding. Commonly known surgical treatments for DU include PD, gastrointestinal reconstruction with ulcer resection, and Dubois surgery [15,16]. However, because the ulcer in our case was shallow and had not spread to major blood vessels or the pancreas, we were able to use the less invasive surgical approach outlined above and had no serious postoperative problems.

## Conclusions

We successfully treated bleeding with massive hematoma and perforation of the duodenum due to an OTSC with a less invasive surgical approach with consideration of the DCS. The concept of DCS and a simple surgical approach could be one of the preferred options to manage critical situations, as in our case. We hope that our case report may help develop strategies for complicated cases of fatal DU bleeding and complications with endoscopic treatment.
